# Widespread rape does not directly appear to increase the overall HIV prevalence in conflict-affected countries: so now what?

**DOI:** 10.1186/1742-7622-5-11

**Published:** 2008-07-29

**Authors:** Aranka Anema, Michel R Joffres, Edward Mills, Paul B Spiegel

**Affiliations:** 1 British Columbia Centre for Excellence in HIV/AIDS, St. Paul's Hospital, Vancouver, Canada; 2 Faculty of Health Sciences, Simon Fraser University, Burnaby, Canada; 3 Public Health and HIV Section, United Nations High Commissioner for Refugees, Geneva, Switzerland

## Abstract

**Background:**

Sub-Saharan Africa (SSA) is severely affected by HIV/AIDS and conflict. Sexual violence as a weapon of war has been associated with concerns about heightened HIV incidence among women. Widespread rape by combatants has been documented in Burundi, Sierra Leone, Rwanda, Democratic Republic of Congo, Liberia, Sudan and Uganda. To examine the assertion that widespread rape may not directly increase HIV prevalence at the population level, we built a model to determine the potential impact of varying scenarios of widespread rape on HIV prevalence in the above seven African countries.

**Discussion:**

Our findings show that even in the most extreme situations, where 15% of the female population was raped, where HIV prevalence among assailants was 8 times the country population prevalence, and where the HIV transmission rate was highest at 4 times the average high rate, widespread rape increased the absolute HIV prevalence of these countries by only 0.023%. These projections support the finding that widespread rape in conflict-affected countries in SSA has not incurred a major direct population-level change in HIV prevalence. However, this must not be interpreted to say that widespread rape does not pose serious problems to women's acquisition of HIV on an individual basis or in specific settings. Furthermore, direct and indirect consequences of sexual violence, such as physical and psychosocial trauma, unwanted pregnancies, and stigma and discrimination cannot be understated.

**Summary:**

The conclusions of this article do not significantly change current practices in the field from an operational perspective. Proper care and treatment must be provided to every survivor of rape regardless of the epidemiological effects of HIV transmission at the population level. Sexual violence must be treated as a protection issue and not solely a reproductive health and psychosocial issue. It is worth publishing data and conclusions that could be misconstrued and may not make much of a programmatic difference in the field. Data, if collected, analysed and interpreted carefully, help to improve our understanding of complicated and nuanced situations. Ultimately, our understanding of what the outcomes of such interventions can achieve will be more realistic. It also helps decision-makers prioritise their funding and interventions.

## Background

Is it worth publishing data and recommendations that could be misconstrued and may not make much of a programmatic difference in the field? Yes.

Data, if collected and analysed correctly and interpreted carefully, help to improve our understanding of complicated and nuanced situations. Even if programmes in the field do not significantly change, our understanding of what the outcomes of such interventions can achieve will be more realistic. It also helps decision-makers prioritise their funding and interventions.

Sub-Saharan Africa (SSA) is overwhelmingly affected by HIV/AIDS [1]. It is also the region with the highest number of armed conflicts worldwide. In 2005, armed conflicts were active in 15 of SSA's 47 countries [2], and resulted in the displacement of approximately 12.5 million people [3]. Armed conflicts have traditionally been considered catalysts for HIV transmission due to associated sexual violence against women, lack of access to preventive and curative heath services, and increased vulnerability and risks incurred by population displacement and food insecurity [4,5]. The use of sexual violence as a weapon of war has been particularly associated with concerns about heightened HIV incidence among women and girls [6,7]. Widespread rape by armed combatants has been documented in Burundi [8], Sierra Leone [9], Rwanda [10], the Democratic Republic of Congo (DRC) [11], Liberia [12], Sudan [13] and Uganda [14]. For the last decade, international organisations have perceived rape as a major cause of HIV transmission in conflict settings, and prioritised public health interventions to mitigate its effects [15-18].

However, empirical research suggests that HIV incidence in armed conflicts may in fact diminish, remain constant or not increase to the same magnitude compared with those countries not in conflict. This may be due to reductions in population mobility, access, and urbanisation [19,20]. A recent systematic review of the seven conflict affected countries found that existing data do not support a link between widespread rape and increased HIV prevalence at the population level [21]. This finding has been met with some criticism from non-governmental organisations and advocacy groups [22], partially because of the perceived HIV-risk associated with rape in these contexts.

This paper builds on Spiegel et al.'s systematic review of HIV in conflict affected countries. To further examine the assertion that widespread rape may not increase HIV prevalence at the population level, we analyse the impact of varying scenarios of widespread rape on HIV prevalence in 7 SSA countries that have undergone conflict.

We built a model to determine the potential impact of varying scenarios of widespread rape on HIV prevalence at the population level in the seven African countries listed above (Table 1). Specifically, we investigated how the total population HIV prevalence would be affected if 1%, 5%, 10% and 15% of the female population aged 5–49 years were raped in each country. We looked at extreme scenarios, by assuming low, intermediate and high estimates of HIV prevalence and transmission rates among male assailants. We assumed that assailants of widespread rape were armed combatants. Given the absence of seroprevalence data for armed combatants in the countries under study, we assumed the HIV prevalence among assailants was commensurate to that of national militaries in SSA. To take into account potential higher prevalence in selected groups (e.g. HIV used as a weapon), we multiplied the baseline population prevalence by 2, 4 and 8. Primary outcomes of our analysis included absolute and relative increases in population level HIV prevalence, per different rape prevalence estimates, for each country, and for age groups 5–49 years. To be conservative in our estimates, we decided to use the 5–49 year age group compared with the 15–49 year group because rape in conflict has been reported among girls less than 15 years of age.

**Table 1:** Estimated increase in prevalence in HIV due to different rates of sexual assault in women 5–49 yrs, by selected countries.

Country	Current HIV/AIDS in Population	Current HIV Prev Total Pop	Women at Risk (5–49 yrs) Total Pop	Different Estimates of Rape	Women at HIV Risk due to Rape	HIV Estimates in Assailant	Transmission Rate	New HIV cases	Increase in HIV Prevalence in Total Population	
	N	%	N	%	N	%	%	N	Absolute %	Relative %

Burundi	150,000	1.85	2,949,494	1	28,948	1.85	0.28	1	0.000	0.00
			8,090,068	5	144,740	3.71	0.8	43	0.001	0.03
				10	289,481	7.42	1.6	344	0.004	0.23
				15	434,221	14.84	3.2	2,062	0.025	1.37
DRC	1,000,000	1.6	23,140,381	1	227,711	1.6	0.28	10	0.000	0.00
			62,660,551	5	1,138,554	3.19	0.8	291	0.000	0.03
				10	2,277,108	6.38	1.6	2,326	0.004	0.23
				15	3,415,663	12.77	3.2	13,955	0.022	1.4
RWANDA	190,000	1.97	3,595,574	1	35,247	1.97	0.28	2	0.000	0.00
			9,638,170	5	176,235	3.94	0.8	56	0.001	0.03
				10	352,469	7.89	1.6	445	0.005	0.23
				15	528,704	15.77	3.2	2,668	0.028	1.4
SIERRA LEONE	48,000	0.8	2,223,610	1	22,058	0.8	0.28	0	0.000	0.00
			6,005,250	5	110,292	1.6	0.8	14	0.000	0.03
				10	220,584	3.2	1.6	113	0.002	0.24
				15	330,876	6.39	3.2	677	0.011	1.41
SOMALIA	44,000	0.5	3,230,922	1	32,149	0.5	0.28	0	0.000	0.00
			8,863,338	5	160,744	0.99	0.8	13	0.000	0.03
				10	321,488	1.99	1.6	102	0.001	0.23
				15	482,232	3.97	3.2	613	0.007	1.39
SUDAN	350,000	0.85	14,310,576	1	141,891	0.85	0.28	3	0.000	0.00
			41,236,378	5	709,456	1.7	0.8	96	0.000	0.03
				10	1,418,911	3.4	1.6	771	0.002	0.22
				15	2,128,367	6.79	3.2	4,625	0.011	1.32
UGANDA	1,000,000	3.42	10,614,367	1	102,509	3.42	0.28	10	0.000	0.00
			29,206,503	5	512,547	6.85	0.8	281	0.001	0.03
				10	1,025,094	13.7	1.6	2,246	0.008	0.22
				15	1,537,641	27.39	3.2	13,478	0.046	1.35
TOTAL	2,782,000	1.68	60,064,924	1	590,565	1.68	0.28	28	0.000	0.00
			165,700,258	5	2,952,824	3.36	0.8	793	0.000	0.03
				10	5,905,647	6.72	1.6	6,346	0.004	0.23
				15	8,858,471	13.43	3.2	38,074	0.023	1.37

We obtained annual population estimates for the countries under study from United States Census Bureau's International Data Base [23]. These population estimates are based on all residents of nations within SSA regardless of legal status or citizenship, and represent a compendium of data on population, fertility, mortality, contraceptive use, and related demographic topics provided by the US Census Bureau [24]. High, low and mid-range estimates of national HIV prevalence estimates for women aged 5–49 years were obtained from UNAIDS/WHO's country-specific end-2006 Epidemiological Fact Sheets for HIV/AIDS and Sexually Transmitted Infections [25].

We determined the number of women at risk of becoming newly infected with HIV from rape by subtracting the estimated number of HIV prevalent cases from the total adult female population. The lower population prevalence was used as a conservative estimate. To account for varying heterosexual transmission rates according to disease stage, multiple rapes, presence of sexually transmitted infections, and other confounding factors, we assumed low, intermediate and high rates of HIV transmission. On the low end, we assumed an HIV transmission rate of 0.0028, which is the acknowledged rate for HIV heterosexual HIV transmission among asymptomatic and symptomatic individuals (i.e. >500 to >200 CD4+ counts). On the upper end, we assumed a rate of 0.008, the rate of heterosexual transmission among individuals with acute and recent primary HIV infection [26]. This upper rate was then multiplied two and four times to take into account potential factors associated with higher rates of transmission, such as genital and/or rectal trauma associated with rape by multiple assailants [27]. The number of women at risk of HIV transmission is the product of the total population of women in this age-group by the probability of rape, taking into account those who are already HIV positive. The number of newly infected women is, therefore, the product of the number of women at risk by the probability of the assailant being positive and the probability of transmission. The absolute increase in prevalence is the number of newly infected women divided by the total population. The relative increase in the total population is therefore this rate divided by the country specific rate (Table 1).

## Discussion

Our findings show that even in the most extreme situation, where 15% of the female adult population level was raped, where HIV prevalence among assailants was 8 times the country population prevalence, and where the HIV transmission rate was highest at 4 times the average high rate, widespread rape increased the absolute HIV prevalence of these countries by only 0.023% (range: 0.007–0.046%). These projections support the finding that widespread rape in conflict affected countries in SSA has not incurred a direct major population-level change in HIV prevalence [21].

Our lowest (0%) or intermediary (0.004%) estimates may, in fact, be most representative of the actual situation. Little information exists about HIV prevalence among armed combatants in conflict affected SSA countries. UNAIDS has previously assumed that rates of HIV among armed combatants is 2 to 5 times higher than among civilian populations [28]. However, a recent study now suggests that only long-serving soldiers and soldiers belonging to less disciplined forces may be at increased risk of infection [29]. HIV prevalence in militaries may be lower than previously assumed due to the common military practice of recruiting young men from low-prevalence rural areas, to screening out HIV-positive individuals through routine testing, and to stationing soldiers in remote areas with limited mobility and remuneration [30].

### So now what?

Risk perception is influenced by a complex interaction between emotional and rational judgements of a situation [31], and psychological, social, cultural and political factors [32]. Elevated feelings of dread combined with unknown risk tend to increase people's perception of risk. This has been demonstrated in the context of blood transfusions, where perceived risk of HIV transmission is high, in spite of the actual risk being quite low when blood is properly screened [33]. The growing body of empirical evidence suggests the international community's perceived risk about the impact of widespread rape on HIV prevalence overestimated the actual risk. Our results provide a rational basis for interpreting the actual risk of direct HIV transmission from widespread rape in conflict affected SSA countries.

Our findings must not be interpreted to say that widespread rape in conflict affected countries does not pose a serious problem to women's acquisition of HIV on an individual basis or in specific settings. Although the increase in total prevalence is small compared with the overall population, it is horrific that tens of thousands of women acquire HIV from sexual violence during conflict. This constitutes egregious human rights violations and is a humanitarian crisis. Furthermore, the direct and indirect consequences of sexual violence, such as physical and psychosocial trauma, unwanted pregnancies, and stigma and discrimination cannot be understated. The relative increase in HIV transmission adds a further dimension of human suffering and health care burden. It increases individual's needs for physical and psychological care, including anti-retroviral therapy. Clearly, for a myriad of reasons, the protection of women and girls in conflict settings remains of paramount importance. Zero tolerance of sexual violence must be an international priority.

The consequences of conflict and rape are long lasting. One long term sequela of rape is its association with sexual risk behaviour and with HIV and other sexually transmitted infections [34-36]. Although difficult to study and quantify, it has been hypothesised that the indirect effects of conflict and rape may ultimately cause women and children to increase their vulnerability and risk behaviour that could ultimately end in their becoming HIV positive. Various forms of exposure to trauma in childhood might result in increased use of violence, including sexual violence, in later years as well as sexual risk-taking [37]. However, it is unclear how these indirect affects of rape would affect the HIV prevalence at the population level.

There are several possible explanations for lower HIV rates than initially considered. These include the reduction in social mobility that occurs during times of conflict, reduced number of sexual partners outside of the rape scenarios, and possibly lower HIV rates amongst the assailants than previously believed. Many of the men who rape are young, live in rural settings, and may have a low prevalence of HIV [38]. However, perhaps the most important factor for the low change in HIV prevalence rates at the population level may be the relatively low transmission rates of HIV infection through sexual intercourse. This is in stark contrast to repeated and long-term exposure that often occurs in concurrent sexual relationships; the latter has shown to increase the likelihood of transmission at an individual and population level given the multiple exposures and interconnecting networks [39].

The conclusions of this article do not have a significant impact on current practices in the field from an operational perspective. Proper care and treatment must be provided to every survivor of rape regardless of the epidemiological effects of HIV transmission at the population level [40-43]. Sexual violence must be treated as a protection issue and not solely a reproductive health and psychosocial issue [40,41,44]. This is regardless if the rape occurs in conflict or from a domestic partner. In fact, rape perpetrated by a stranger reflects only a small proportion of women's experiences of coerced sex which is more commonly perpetrated by known persons such as within marriages, dating relationships and families [45].

Although there is a growing acknowledgement among international organisations about the collective responsibility to protect civilians in conflict settings [46], Governments, United Nations agencies and non-governmental organisations must systematically mainstream protection of women and girls in all of their field-based operations as soon as the emergency begins. The presence of international expatriate staff has some protective impact on civilians by deterring belligerents, creating confidence in communities and attracting global attention to crises [47]. However, organisations providing humanitarian protection in conflict settings would benefit from implementing existing standards and guidelines to ensure adequate responsive and remedial actions are taken to prevent sexual violence [41,40,48-50] and to ensure appropriate protection, health and psychosocial services are in place (e.g. HIV post-exposure prophylaxis, treatment of sexually transmitted infections, and psychosocial support). International law may be useful for setting standards and goals that frame a mission's rules of engagement and entry agreements [42]. Sustainable behaviour change requires national support for humanitarian ethics and its duties, including the adoption and ratification of International Humanitarian Law [51].

There is a need for the international community to better understand the drivers of sexual violence against women in armed conflicts. This is critical in countries such as the DRC, where rape at the hands of government and guerrilla armed combatants is reportedly increasing in numbers and magnitude of violence [52], leaving many survivors debilitated with genital fistulae [53]. Increased attention should be undertaken to promote HIV prevention practices among armed combatants. In response to the problem of troops being both victims and transmitters of the virus, the UN Security Council adopted Resolution 1308 that calls for national strategies to address the spread of AIDS among uniformed services, including through awareness raising and training among their ranks [43]. UNAIDS and the UN Department of Peacekeeping Operations are working with national authorities to mainstream HIV/AIDS programs in all UN peacekeeping missions. AIDS advisors have been placed in 29 SSA countries, including those with current or recent conflict such as Burundi, Côte d'Ivoire, DRC, Ethiopia, Eritrea, Liberia, Sierra Leone and Sudan [54].

Our findings also draw attention to the need for improved surveillance of sexual violence and its impact on HIV epidemiology. Humanitarian organisations, including UN organisations, as well as the media have sometimes published inaccurate or misleading HIV information about conflict-affected populations [55]. Poor survey methods, studies restricted to urban centres with elevated prevalence, and biased analyses of findings have contributed to the assumption that HIV transmission dramatically increases in conflict settings [21]. Organisations conducting epidemiological assessments of, and reporting on HIV seroprevalence in these contexts may benefit from standardised epidemiological training to ensure their surveillance methodologies are scientifically sound and reproducible, and their reported data are more precise [56].

## Summary

Some persons will justifiably be concerned that publishing such an article will do harm to all of the important efforts that have occurred to ensure that sexual exploitation and violence are recognised as the most basic of human rights violations and essential interventions must be provided to all survivors of such heinous acts in all contexts. Since widespread rape in conflict situations does not appear to directly increase HIV prevalence at the population level, should donors and other decision makers decide to put their limited funds, personnel and interventions towards other groups and programmes in different contexts that may have a larger public health affect? They may and are free to do so. However, we would strongly recommend against it due to the reasons stated above. Despite the uncomfortable findings of this article and the possibility that people may misinterpret or correctly interpret the findings of this article and decide to prioritise programmes other than sexual violence, we still believe it is important to publish such a paper. We dread the possibility that some journalist may try to grab the headlines by writing "Rape does not increase HIV". However, that concern does not justify not having an open, honest, intelligent and nuanced discussion about rape and its affect on HIV transmission at the individual and population levels.

## Competing interests

The authors declare that they have no competing interests.

## Authors' contributions

AA undertook the modeling and drafted the manuscript. MRJ underook the modeling and commented on the manuscript. EM undertook the background research, helped to draft the manuscript, and participated in the revisions of the manuscript. PS conceptualised the design of manuscript, helped to draft the manuscript, and participated in the revisions of the manuscript. All authors read and approved the final manuscript.
